# A Peptide Derived from G_0_/G_1_ Switch Gene 2 Acts as Noncompetitive Inhibitor of Adipose Triglyceride Lipase*

**DOI:** 10.1074/jbc.M114.602599

**Published:** 2014-09-25

**Authors:** Ines K. Cerk, Barbara Salzburger, Andras Boeszoermenyi, Christoph Heier, Christoph Pillip, Matthias Romauch, Martina Schweiger, Irina Cornaciu, Achim Lass, Robert Zimmermann, Rudolf Zechner, Monika Oberer

**Affiliations:** From the Institute of Molecular Biosciences, University of Graz, 8010 Graz, Austria

**Keywords:** Adipose Triglyceride Lipase (ATGL), Enzyme Inhibitor, Lipid, Lipid Metabolism, Lipolysis, Peptides, Atglistatin, G0/G1 Switch Gene 2 (G0S2), hGW2052, Noncompetitive Inhibition

## Abstract

The protein G_0_/G_1_ switch gene 2 (G0S2) is a small basic protein that functions as an endogenous inhibitor of adipose triglyceride lipase (ATGL), a key enzyme in intracellular lipolysis. In this study, we identified a short sequence covering residues Lys-20 to Ala-52 in G0S2 that is still fully capable of inhibiting mouse and human ATGL. We found that a synthetic peptide corresponding to this region inhibits ATGL in a noncompetitive manner in the nanomolar range. This peptide is highly selective for ATGL and does not inhibit other lipases, including hormone-sensitive lipase, monoacylglycerol lipase, lipoprotein lipase, and patatin domain-containing phospholipases 6 and 7. Because increased lipolysis is linked to the development of metabolic disorders, the inhibition of ATGL by G0S2-derived peptides may represent a novel therapeutic tool to modulate lipolysis.

## Teaser

**Background:** G0S2 acts as an endogenous inhibitor of ATGL.

**Results:** We designed a short peptide that is active in the nanomolar range. The G0S2-derived peptide inhibits ATGL activity in a noncompetitive manner.

**Conclusion:** G0S2-derived peptides act as potent and specific inhibitors of ATGL.

**Significance:** G0S2-derived peptides provide a novel tool to modulate ATGL activity.

## Introduction

Most organisms store excess energy as neutral and inert triacylglycerols (TGs)^2^ to ensure the availability of energy in the event of demand. These energy stores can be rapidly mobilized in a process termed lipolysis that generates glycerol and fatty acids (FAs). Glycerol can subsequently be channeled toward glycolysis or TG synthesis. FAs serve as energy substrate, represent signaling molecules, and can be used for anabolic reactions. FA metabolism is closely linked to metabolic diseases. Excess FA concentrations in the circulation lead to deleterious effects on the organism, including ectopic lipid accumulation, insulin resistance, and inflammation, summarized in the term lipotoxicity (1–4). Consequently, TG hydrolysis and synthesis are tightly regulated processes that ensure an adequate supply of FAs to oxidative tissues while avoiding FA overload in ectopic tissues (5, 6). Understanding the mechanisms that regulate lipolysis and concomitantly the release of FAs are of fundamental interest.

Adipose triglyceride lipase (ATGL) hydrolyzes TGs into diacylglycerols and FAs and is required for efficient intracellular lipolysis (7). Global inactivation of ATGL activity leads to massive TG accumulation in all tissues, including the heart of *Atgl* knock-out mice (8). Accordingly, patients with ATGL deficiency suffer from cardiac and skeletal muscle myopathy and require heart transplants at a relatively young age (9). However, genetic and small molecule-mediated inactivation of ATGL is also associated with beneficial effects on plasma lipid parameters and insulin sensitivity; *Atgl*-KO mice and WT mice treated with the small molecule inhibitor Atglistatin show reduced plasma FA and TG concentrations (8, 10). Consequently, *Atgl*-KO mice display improved glucose tolerance and insulin sensitivity (8). Recently, it has been reported that *Atgl*-KO mice are also resistant to the development of cancer-associated cachexia, a multifactorial syndrome characterized by ongoing loss of skeletal muscle and fat mass (11).

Extensive research on the physiological role of ATGL has identified complex regulatory mechanisms for its activity on the transcriptional and post-translational level (12, 13). Direct protein-protein interaction of ATGL with the protein comparative gene identification 58 (CGI-58) increases ATGL activity up to 20-fold (14). Inhibition of ATGL due to direct interaction with the small, 103-amino acid protein G_0_/G_1_ switch gene 2 (G0S2) was discovered in 2010 and confirmed using human and murine proteins (15–21). The highest G0S2 expression is found in adipose tissues, but it is also present in other tissues, including the liver and the heart (20, 22, 23). To date, no mutation in human G0S2 has been reported. Global and liver-specific *G0s2*-KO mouse models were generated very recently and display decreased adipose tissue mass (24, 25), enhanced lipolysis in adipose tissue, and a decrease in hepatic TG content (26). Global and adipose tissue-specific overexpression of G0S2 leads to increased fat mass, overall reduction in lipolytic activity, and fatty liver (16, 19, 26).

Activation of ATGL by CGI-58 is considered as fasting response upon β-adrenergic stimulation and phosphorylation of PKA (12). Recent studies have strengthened the role of G0S2 as the so-called master regulator of basal and stimulated lipolysis (27). mRNA levels of G0S2 respond strongly to hormonal stimuli, yet the dose-dependent inhibition of ATGL by G0S2 itself is reported to be independent of phosphorylation events (18, 20, 21, 28, 29). Thus, regulation of the expression levels of G0S2, and consequently the protein concentration, resembles a potent mean to regulate lipid mobilization. Elegant studies of Zhang *et al.* (26) demonstrated an important physiological link of G0S2 in regulating adipose tissue lipolysis and liver energy homeostasis. Accordingly, G0S2 mRNA levels are regulated differently in adipose tissue and the liver. During feeding, G0S2 mRNA is detected only in low levels in the liver and at high levels in WAT where it promotes the storage of lipids. Upon fasting, G0S2 concentrations in WAT decrease, promoting lipolysis and WAT-derived FA transport to the liver and other organs. Simultaneously, the increase in liver G0S2 expression inhibits the degradation of endogenous TG stores and thus can promote fasting-induced liver steatosis (26). Consequently, tissue-specific regulation of G0S2 provides a mechanism controlling TG storage in different organs depending on the metabolic state. Notably, G0S2 is reduced in WAT of diabetic individuals (30) suggesting that changes in G0S2 expression contribute to dysregulated lipolysis observed in diabetic patients. Other physiological processes have also been associated with G0S2 and include adipogenesis (24), proliferation (31, 32), apoptosis (33), immune regulation (34–37), oxidative phosphorylation (38), tumor suppression, and cancer (33, 39–41), but they have not been studied extensively. Our goal is to identify peptide inhibitors for ATGL based on the physiological inhibition by G0S2. To do so, we first have to understand the underlying mechanisms of ATGL inhibition by G0S2. Previous reports suggest that a truncated variant of G0S2 encompassing the residues Met-1–Gln-73 interacts with ATGL and inhibits the enzyme. Another variant lacking an extensive central region (residues Tyr-27–Leu-42) failed to immunoprecipitate with ATGL and had no inhibitory activity (20). These results already indicated that the N-terminal portion of G0S2 is essential for ATGL inhibition. However, deletion of the central region of G0S2 might have also resulted in loss-of-function due to complete disruption of the protein fold. Consequently, we first delineated the minimal sequence boundaries for a biologically active, truncated G0S2 variant. Furthermore, we describe a synthetic inhibitory peptide containing a sequence stretch derived from G0S2, and we kinetically characterize the mode of inhibition by this peptide. Our study provides first insights into the mode of inhibition and opens avenues to achieve tissue-specific inhibition of ATGL based on a synthetic peptide.

## EXPERIMENTAL PROCEDURES

### 

#### 

##### Materials

[9,10-^3^H]Triolein was obtained from PerkinElmer Life Sciences. Triolein, phosphatidylcholine, phosphatidylinositol, 1(*rac*)-oleoylglycerol, oleoyl-CoA, and free glycerol detection reagents were purchased from Sigma. 1-Oleoyl-2-hydroxy-*sn*-glycero-3-phosphocholine was purchased from Avanti Polar Lipids Inc., Alabaster, AL, and the NEFA kit was from WAKO Diagnostics, Neuss, Germany. Hi76-0079 obtained from Novo Nordisk, Denmark, Atglistatin was a generous gift from R. Breinbauer (Graz University of Technology, Austria). The protein assay kit was obtained from Bio-Rad; Thermo Scientific, Rockford, IL was the source for the Pierce® BCA protein assay kit. The synthetic peptides were synthesized by Peptide Specialty Laboratories GmbH, Heidelberg, Germany.

##### Cloning of Recombinant Proteins

Human *G0S*2 (*hG0S2*), including the complete open reading frame, was amplified from cDNA by PCR using Phusion^TM^ polymerase (New England Biolabs, Ipswich, MA) and primers containing endonuclease cleavage sites for subsequent cloning into a modified pSUMO vector (kindly provided by Prof. Christoph D. Lima, Sloan-Kettering Institute) with a tobacco etch virus (TEV) protease cleavage site for tag removal (*his_6_-smt-hG0S2*). N-terminal truncations were generated by PCR using primers flanking the respective sequence of *hG0S2* and containing endonuclease cleavage sites for insertion into the target vector. C-terminal truncations of *hG0S2* were obtained by introducing stop codons using the QuikChange® site-directed mutagenesis kit (Agilent Technologies, Santa Clara, CA). Primers used are listed in Table 1. Sequences containing the coding sequences of mouse *Cgi-58* and mouse *Atgl* were inserted in pSUMO (see above) and pASK-IBA5plus (IBA, Goettingen, Germany) vectors, respectively, as described previously (13, 42). pcDNA4/HisMax vectors (Invitrogen) encoding mouse *Atgl*, patatin domain-containing proteins 6 and 7 (*mPnpla6* and *mPnpla7*), hormone-sensitive lipase (*Hsl*), and monoacylglycerol lipase (*Mgl*) coding sequences were generated as described previously (7, 43, 44).

**TABLE 1:** **Primers used for cloning of *hG0S2***

Name	Primer forward (5′–3′)	Primer reverse (5′–3′)
hG0S2_1-103	GGCGGATCCATGGAAACGGTCCAGG	CCGCTCGAGCTAAGAGGCGTGCTGC
hG0S2_10-103	GACCGGATCCCTGGCCAAGGAGATGATGG	CCGCTCGAGCTAAGAGGCGTGCTGC
hG0S2_20-103	GGAGCCGGATCCAAGGGGAAGATGGTGAAGC	CCGCTCGAGCTAAGAGGCGTGCTGC
hG0S2_22-103	GGCGCCGGATCCAAGATGGTGAAGCTGTACG	CCGCTCGAGCTAAGAGGCGTGCTGC
hG0S2_23-103	GGAGCCGGATCCATGGTGAAGCTGTACGTGC	CCGCTCGAGCTAAGAGGCGTGCTGC
hG0S2_24–103	GGAGTCGGATCCGTGAAGCTGTACGTGCTGG	CCGCTCGAGCTAAGAGGCGTGCTGC
hG0S2_27-103	GCACAGGATCCTACGTGCTGGGCAGC	CCGCTCGAGCTAAGAGGCGTGCTGC
hG0S2_28–103	GGTATAGGATCCGTGCTGGGCAGCGTGC	CCGCTCGAGCTAAGAGGCGTGCTGC
hG0S2_29-103	GGTATAGGATCCCTGGGCAGCGTGCTGG	CCGCTCGAGCTAAGAGGCGTGCTGC
hG0S2_30-103	GTACAGGATCCGGCAGCGTGCTGGC	CCGCTCGAGCTAAGAGGCGTGCTGC

**Site-directed mutagenesis primer**		
hG0S2_52	GCAGCCCCTTCACGGCCTAAAGACGTCTGCGGGACC	GGTCCCGCAGACGTCTTTAGGCCGTGAAGGGGCTGC
hG0S2_46	CCTGATGGAGACTGTGTGAAGCCCCTTCACGGCCGCCAGACG	CGTCTGGCGGCCGTGAAGGGGCTTCACACAGTCTCCATCAGG
hG0S2_45	CCTGATGGAGACTTAGTGCAGCCCCTTCACG	CGTGAAGGGGCTGCACTAAGTCTCCATCAGG
hG0S2_44	GCTCGGCCTGATGGAGTAAGTGTGCAGCCCCTTCACG	CGTGAAGGGGCTGCACACTTACTCCATCAGGCCGAGC
hG0S2_43	GGTGCTCGGCCTGATGTAGACTGTGTGCAGC	GCTGCACACAGTCTACATCAGGCCGAGCACC
hG0S2_42	GGTGCTCGGCCTGTAAGAGACTGTGTGC	GCACACAGTCTCTTACAGGCCGAGCACC
hG0S2_41	CGTGGTGCTCGGCTAGATGGAGACTGTGTGC	GCACACAGTCTCCATCTAGCCGAGCACCACG
hG0S2_40	CCTCTTCGGCGTGGTGCTCTAACTGATGGAGACTGTGTGC	GCACACAGTCTCCATCAGTTAGAGCACCACGCCGAAGAGG
hG0S2_39	CCTCTTCGGCGTGGTGTAAGGCCTGATGGAGACTGTGTGC	GCACACAGTCTCCATCAGGCCTTACACCACGCCGAAGAGG

##### Bacterial Expression of Recombinant Proteins and Preparation of Cell Extracts

Human *his_6_-smt-G0S2* constructs were transformed into *Escherichia coli* BL21(DE3) CodonPlus® cells (Stratagene, La Jolla, CA). Cultures were grown at 37 °C on selective LB medium containing 40 μg/ml kanamycin to an *A*_600_ of 0.5. Expression was induced by the addition of 0.5 mm isopropyl-β-d-thiogalactopyranoside at 30 °C. After 15 h of induction, cells were harvested, resuspended in sucrose solution (250 mm sucrose, 1 mm EDTA, 1 mm DTT, 20 μg/ml leupeptin, 2 μg/ml antipain, 1 μg/ml pepstatin, pH 7.0), and disrupted by sonication (SONOPLUS ultrasonic homogenizer HD 2070, Bandelin, Berlin, Germany) on ice. After centrifugation at 15,000 × *g* for 20 min at 4 °C, the supernatants were collected. Protein concentrations were determined as described below. Expression of the murine ATGL-Strep fusion (Strep-mATGL) and His_6_-Smt-mCGI-58 in *E. coli* is described in Refs. 13 and 42, respectively.

##### Expression of Recombinant Proteins in COS-7 Cells and Preparation of Cell Lysates

Simian SV-40 transformed monkey kidney cells (COS-7 ATCC CRL-1651) were cultured in DMEM (Invitrogen) containing 10% FCS (Sigma) under standard conditions (95% humidified atmosphere, 37 °C, 5% CO_2_). Cells were transiently transfected with pcDNA4/HisMax plasmid coding for *mAtgl*, *mPnpla6*, *mPnpla7*, *mHsl*, *mMgl*, or β-galactosidase (*lacZ*) using Metafectene^TM^ (Biontex GmbH, Munich, Germany) as described (7, 43, 44). Cells were harvested 48 h after transfection. For the preparation of cell lysates, cells were resuspended in sucrose solution (see above) and disrupted by sonication (SONOPLUS ultrasonic homogenizer HD 2070) on ice. Nuclei and unbroken cells were removed by centrifugation at 1,000 × *g* for 10 min at 4 °C. Protein concentrations were determined as described below.

##### Purification of Recombinant Murine CGI-58

Purification of the His_6_-Smt-tagged mCGI-58 was performed via immobilized metal ion affinity chromatography. For the preparation of cell extracts, *E. coli* cells were resuspended in buffer A (20 mm Tris-HCl, 500 mm NaCl, 0.1% IgePal CA-630, 30 mm imidazole, 1 mm tris(2-carboxyelthyl)phosphine hydrochloride (TCEP), 1 mm benzamidine, 0.1 mm PMSF, pH 7.8) and disrupted by sonication (SONOPLUS ultrasonic homogenizer HD 2070) on ice. After centrifugation (15,000 × *g*, 20 min, 4 °C), the soluble fraction of mCGI-58 was isolated using a 5-ml HisTrap^TM^ FF column (GE Healthcare). The protein was eluted in 10 column volumes of a 0–100% buffer B gradient (20 mm Tris-HCl, 500 mm NaCl, 250 mm imidazole 10% glycerol, 1 mm TCEP, 1 mm benzamidine, pH 7.8). Fractions containing mCGI-58 were subjected to an additional size exclusion chromatography step using a Superdex 200 column (GE Healthcare) and 20 mm Tris-HCl, 200 mm NaCl, 10% glycerol, 1 mm EDTA, 1 mm TCEP, pH 7.8, as mobile phase. Protein concentration was determined via the absorption at 280 nm.

##### Purification of Recombinant Human G0S2

*E. coli* cells containing the His_6_-Smt-tagged hG0S2 (His_6_-Smt-hG0S2) were resuspended in buffer A (adjusted to pH 7.5) and disrupted by sonication (SONOPLUS ultrasonic homogenizer HD 2070) on ice. After centrifugation at 15,000 × *g* for 20 min at 4 °C, His_6_-Smt-hG0S2 was purified from the cellular extract by affinity chromatography using a 5-ml HisTrap^TM^ FF column (GE Healthcare). Purified recombinant protein was eluted in 10 column volumes of a 0–100% buffer B (adjusted to pH 7.5) gradient. Next, the His_6_-Smt tag was cleaved off by the addition of 1 mm EDTA and TEV protease in a ratio of 1:100 (protein/TEV protease). The cleavage was performed overnight at 4 °C. Cleaved hG0S2 was isolated by size exclusion chromatography using a Superdex 200 column (GE Healthcare) and 15 mm Na_2_HPO_4_, 5 mm KH_2_PO_4_, 300 mm NaCl, 1 mm EDTA, 1 mm TCEP, pH 7.0, as mobile phase. Pooled fractions containing cleaved hG0S2 were dialyzed (3,500 Da cutoff) extensively against MilliQ® (Millipore Corp., Billerica, MA) water at 4 °C before lyophilization. The lyophilized powder was dissolved in 100% DMSO, and the concentration was determined with Pierce® BCA protein assay kit as described below.

##### Preparation of Tissue Homogenates

Adipose tissue samples from overnight fasted wild-type and *Atgl*-KO mice were washed in PBS (137 mm NaCl, 2.7 mm KCl, 10 mm Na_2_HPO_4_, 2 mm KH_2_PO_4_) containing 1 mm EDTA and homogenized on ice in sucrose solution (see above) using an Ultra Turrax (IKA, Staufen, Germany). After centrifugation at 10,000 × *g* for 20 min at 4 °C, the lipid-free infranatant was collected, and the protein content was determined as described below.

##### Preparation of Synthetic Peptide

Synthetic peptides were purchased from Peptide Specialty Laboratories GmbH, Heidelberg, Germany. The sequences of the inhibitory peptide hGW2052 and the control peptide were WKGKMVKLYVLGSVLALFGVVLGLMETVCSPFTA and VDSADAGGGSGWLTGWLPTWCP, respectively. The peptides were dissolved in 100% DMSO, and concentrations were determined via the absorbance at 280 nm. The final DMSO concentration in the activity assay was ≤1%. The control peptide was prepared the same way.

##### TG Hydrolase Activity Assay

TG hydrolase activity assay was performed as described (45). To screen for the inhibitory capacity of various hG0S2 truncations, 30 μg of protein of cell lysates were incubated with the [^3^H]triolein substrate together with 50 μg of cell extract of Strep-mATGL (or COS-7-mATGL) and 2.5 μg of purified mCGI-58. As a control, lysates of the His_6_-Smt tag were incubated under the same conditions. To determine the enzyme kinetics and the inhibitory mechanism of the hGW2052 peptide, the assay was performed with minor modifications. TG substrate was prepared with 1.67 mm triolein, 10 μCi/ml [9,10-^3^H]triolein (PerkinElmer Life Sciences), and 190 μm phosphatidylcholine/phosphatidylinositol (3:1) (Sigma) and diluted to the respective concentrations after sonication. Assays with CGI-58 co-activated ATGL were performed on a smaller scale. Briefly, cell lysates of Strep-mATGL (25 μg of total protein content) were mixed with 2.5 μg of purified mCGI-58 and 1% of the synthetic peptide hGW2052, control peptide, or DMSO in a total volume of 25 μl of sucrose solution (see above) and were incubated with 25 μl of [^3^H]triolein substrate. Lysates using LacZ-expressing cells were used as controls. The reaction was terminated by adding 650 μl of methanol/chloroform/heptane (10:9:7) and 200 μl of 100 mm potassium carbonate buffer, pH 10.5 (adjusted with boric acid). The radioactivity in 200 μl of the upper phase (450 μl in total) was determined by liquid scintillation counting. Nonlinear regression analysis for inhibitor kinetics was carried out using GraphPad Prism 5 (GraphPad Software Inc., La Jolla, CA). TG hydrolase activity of murine HSL (expressed in COS-7 cells) and purified bovine lipoprotein lipase (Sigma) was determined under the same conditions.

##### Monoacylglycerol Hydrolase Activity Assay

Monoacylglycerol hydrolase activity of murine MGL was determined as described (46). In brief, 1% of hGW2052, the control peptide, or DMSO was added to 2 μg of lysate of COS-7 cells overexpressing murine MGL in a total volume of 10 μl. The mixture was incubated with 100 μl of *rac*-1-(3)-oleoylglycerol (Sigma) as substrate for 20 min at 37 °C. After incubation, the reaction was terminated by adding 100 μl of chloroform. After vigorous mixing using a vortex and centrifugation at 10,000 × *g* for 5 min, the glycerol release (into the upper aqueous phase) was determined using a commercial kit (free glycerol Reagent, Sigma) according to the manufacturer's protocol.

##### Lysophospholipase Activity Assay

Lysophospholipase activity of PNPLA6 and PNPLA7 was determined as described previously (44). In brief, 1% of the hGW2052 peptide, the control peptide, or DMSO was added to cell lysates of COS-7 cells overexpressing PNPLA6 (15 μg), PNPLA7 (50 μg), and lacZ in a total volume of 50 μl of sucrose solution (see above). The mixtures were incubated with 50 μl of 3 mm substrate 1-oleoyl-2-hydroxy-*sn*-glycero-3-phosphocholine (Avanti Polar Lipids Inc., Alabaster, AL) in a water bath at 37 °C for 20 min. Each reaction was terminated by heat inactivation at 75 °C for 10 min. The released amounts of fatty acids were determined using a commercial assay kit (HR Series NEFA-HR(2), WAKO Diagnostics) according to the manufacturer's protocol.

##### Determination of Protein Concentration

Protein concentrations of cell extracts were determined using the protein assay Kit according to the manufacturer's instructions (Bio-Rad) using BSA as standard. The concentrations of purified proteins were obtained by the absorbance at 280 nm using the NanoDrop® ND-1000 spectrophotometer (PEQLAB Biotechnologie GmbH, Erlangen, Germany). Alternatively, protein concentrations were determined using the Pierce® BCA protein assay kit and BSA as standard according to the manufacturer's protocol (Thermo Scientific).

##### Statistical Analysis

All measurements were performed in triplicates. Data are presented as means ± S.D. Statistical significance was determined by the Student's unpaired *t* test (two-tailed). Group differences were considered statistically significant for *p* < 0.05 (*), *p* < 0.01 (**), and *p* < 0.001 (***).

## RESULTS

### 

#### 

##### The N-terminal Region of G0S2 Is Essential for ATGL Inhibition

**FIGURE 1.**
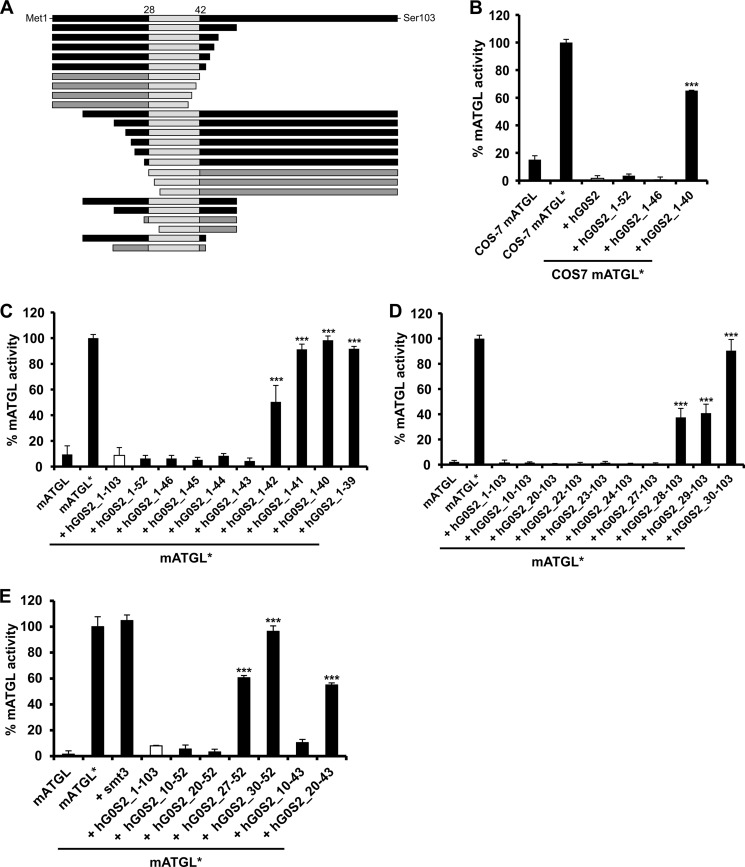
**Minimal sequence requirements of human G0S2 for ATGL inhibition.** *A,* graphical representation of full-length and shortened versions of hG0S2, those capable of inhibiting ATGL activity are illustrated in *black*. The largely hydrophobic sequence stretch from Val-28 to Met-42 is depicted in *light gray. B–E,* activity assays were performed in the presence of CGI-58 as indicated by an asterisk (ATGL*). G0S2 and variants thereof were expressed in *E. coli* and added as cell lysates. Overexpressed mATGL from *E. coli* lysates was used for all assays with the exception of the assay shown in *B. B,* inhibition of TG hydrolase activity of mATGL containing COS-7 cell extracts by WT and C-terminally truncated hG0S2 variants. *C,* inhibition of TG hydrolase activity of mATGL by WT and C-terminally truncated hG0S2 variants. *D,* inhibition of TG hydrolase activity of mATGL by WT hG0S2 and variants with N-terminal deletions. *E,* effect of different N- and C-terminal truncations in hG0S2 on mATGL activity. The fusion tag smt3 is shown as control.

To identify the minimal sequence within G0S2 that is strictly required to exert an inhibitory effect on ATGL activity, we tested different C- and N-terminally shortened versions of human G0S2 (hG0S2) (Fig. 1*A*). As ATGL exhibits very low basal activity, we performed activity assays in the presence of CGI-58 to fully stimulate ATGL. Our results show that C-terminal truncations of G0S2 up to residue Val-46 completely inhibit CGI-58 activated ATGL activity (abbreviated as ATGL* in all figures) in COS-7 lysates (Fig. 1*B*). Inhibition was significantly reduced when hG0S2 was truncated at residue Leu-40 (Fig. 1*B*). These results were reproduced using proteins (ATGL, CGI-58, and G0S2) expressed in *E. coli* (Fig. 1*C*), suggesting that other mammalian proteins are not required for G0S2-mediated inhibition. It also demonstrates that mouse ATGL is also inhibited by human G0S2. Next, we investigated the amino acid residues between Leu-40 and Val-46 and their necessity for ATGL inhibition by G0S2 in more detail. We show that the G0S2 variant Met-1–Met-43 (hG0S2_1–43) harbors the longest C-terminal deletion that still exerts the full inhibitory effect on ATGL (Fig. 1*C*). Screening various N-terminal deletions revealed that truncations of hG0S2 until residue Tyr-27 (hG0S2_27–103) fully inhibit ATGL activity (Fig. 1*D*). Further truncation of the N terminus resulted in significant loss of the inhibitory effect. Truncation of G0S2 after residue Gly-30 resulted in complete loss of the capacity to inhibit ATGL. Next, we tested the effect of a combination of N- and C-terminal truncations of hG0S2. As expected, hG0S2 Leu-10–Ala-52 and Lys-20–Ala-52 fully retained their activity to inhibit ATGL, although hG0S2 Gly-30–Ala-52 had lost all inhibitory activity. Interestingly, neither hG0S2 Tyr-27–Ala-52 nor hG0S2 Lys-20–Met-43 exhibited full inhibitory capacity (Fig. 1*E*), as extrapolation of the findings presented above would have suggested (Fig. 1, *C* and *D*).

##### The Peptide Corresponding to Residues Lys-20 to Ala-52 (hGW2052) from G0S2 Inhibits ATGL in the Nanomolar Range

**FIGURE 2.**
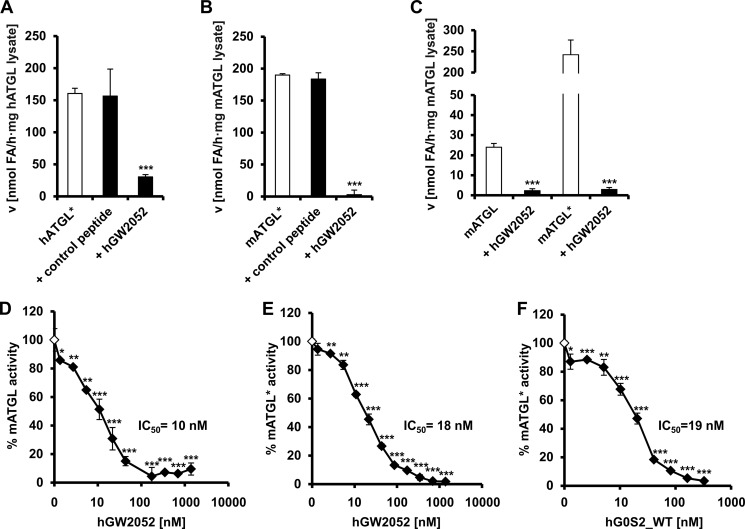
**Inhibition of ATGL by the peptide hGW2052 corresponding to residues Lys-20 to Ala-52 of hG0S2.** Activity assays performed in the presence of CGI-58 are indicated by an asterisk (ATGL*). *A* and *B,* TG hydrolase activity assays of CGI-58 activated ATGL in the absence and in the presence of 1.4 μm hGW2052. A peptide harboring a sequence unrelated to G0S2 served as negative control. *A,* TG hydrolase activity of hATGL* expressed in COS-7 cells. *B,* TG hydrolase activity of mATGL* expressed in *E. coli. C–F,* mATGL used for these assays was overexpressed in *E. coli. C,* inhibition of the TG hydrolase activity of mATGL- and CGI-58-stimulated mATGL by 1.4 μm of the peptide hGW2052. *D,* dose-dependent inhibition of TG hydrolase activity of mATGL. *E,* mCGI-58-stimulated mATGL by the peptide hGW2052. *F,* dose-dependent inhibition of CGI-58-stimulated mATGL by WT hG0S2. Statistical significance was assigned according to the following scheme: *, *p* < 0.05; **, *p* < 0.01; ***, *p* < 0.001.

In our next experiments, we investigated whether synthetic peptides with sequence stretches corresponding to G0S2 are efficient in inhibiting ATGL, and we characterized the peptide-mediated inhibition kinetic of ATGL. Therefore, we designed a synthetic peptide identical to the human G0S2 sequence from Lys-20 to Ala-52 (Fig. 1*A*). A Trp residue was introduced at the N terminus of this peptide to facilitate concentration determination by UV spectroscopy. The resulting peptide was termed hGW2052. In agreement with the overexpressed G0S2 variant hG0S2_20-52 (Fig. 1), the synthetic peptide hGW2052 completely inhibited CGI-58-activated hATGL and CGI-58-activated mATGL expressed in COS-7 and *E. coli* cells, respectively (Fig. 2, *A* and *B*). This indicates that the synthesized peptide adopts a tertiary structure compatible for protein-protein interaction with ATGL. A control peptide was equally treated and did not show an inhibitory effect on ATGL (Fig. 2, *A* and *B*). Next, we tested whether hGW2052 inhibits ATGL also in the absence of CGI-58. Indeed, we found that hGW2052 inhibits ATGL independently of the presence of CGI-58 (Fig. 2*C*). The dose-response experiments of basal ATGL- and CGI-58-stimulated ATGL activity using increasing amounts of hGW2052 revealed half-maximal inhibitory concentrations (IC_50_) of 10 and 18 nm, respectively (Fig. 2, *D* and *E*). In agreement with these results, dose-response experiments using overexpressed, full-length hG0S2 revealed an IC_50_ of 19 nm (Fig. 2*F*).

##### The Peptide hGW2052 Inhibits ATGL in a Noncompetitive Manner

**FIGURE 3.**
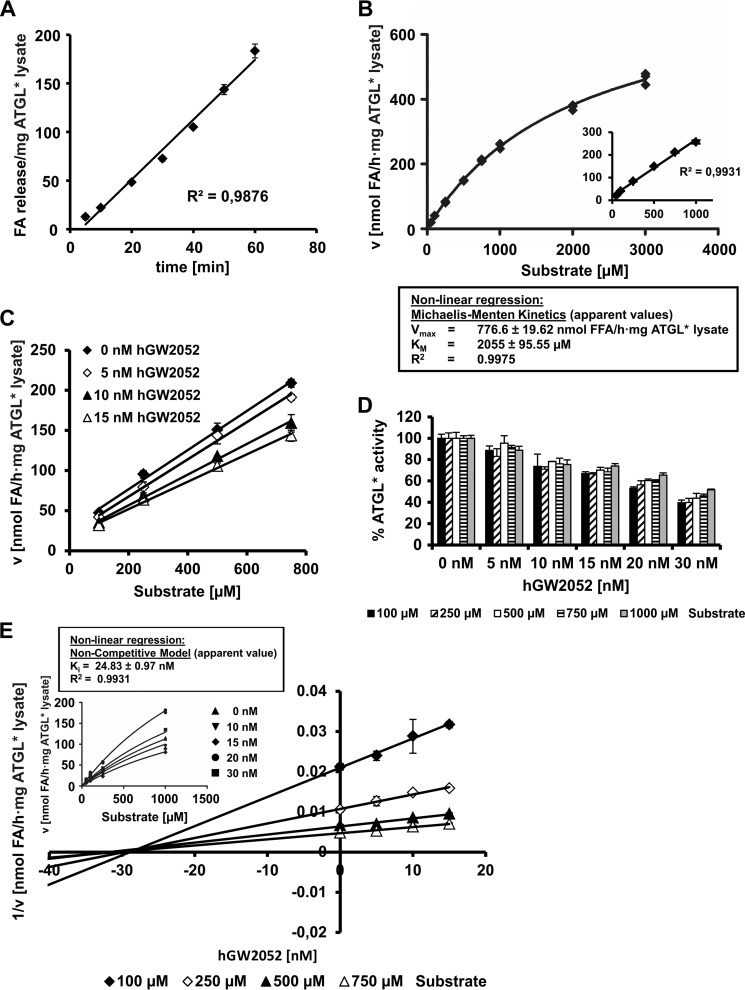
**hGW2052 inhibits ATGL via a noncompetitive mechanism.** All TG hydrolase activity assays in this figure were performed with mATGL expressed in *E. coli* and in the presence of CGI-58 (ATGL*). *A,* time dependence of FA release during TG hydrolase activity assay. *B,* reaction velocity of mATGL lysates depends on the concentration of triolein substrate. *C,* relationship between TG hydrolase reaction rates and substrate concentration (within the linear concentration range) at varying hGW2052 concentrations. *D,* inhibition of mATGL activity in the presence of different hGW2052 concentrations with increasing concentrations of triolein substrate. *E,* Dixon-Plot for kinetic analysis of mATGL inhibition by hGW2052. The reciprocal velocity is plotted against the concentration of hGW2052 at various substrate concentrations. In noncompetitive inhibition, the *lines* converge on the *x* axis with the intersection point giving −*K_i_*. The *inset* shows nonlinear regression analysis (GraphPad Prism 5) using the equation for noncompetitive inhibition.

To investigate the mode of ATGL inhibition by hGW2052, we performed enzyme kinetics. In agreement with previously published data, time course experiments showed a linear increase in FA release of ATGL-containing lysates incubated with an artificial TG substrate for up to 60 min (Fig. 3*A*) (13). Furthermore, a linear increase in apparent enzyme velocity up to 1,000 μm triolein substrate was observed in saturation kinetic experiments in the absence of an inhibitor (Fig. 3*B*). Within that substrate concentration range, we also observed a linear increase in enzyme velocity in the presence of different inhibitor concentrations (Fig. 3, *C* and *D*). Dixon-Plot analysis (47) (plotting 1/*v* against inhibitor concentration at each concentration of triolein substrate) resulted in a set of intersecting lines on the *x* axis compatible with a noncompetitive inhibition mechanism (Fig. 3*E*). Using this method, we determined an apparent *K_i_* of 28 nm for hGW2052. Nonlinear regression analysis (Graph Pad Prism) using a noncompetitive inhibition model was also used to extract kinetic parameters and resulted in an apparent *K_i_* of 25 ± 1 nm, which is in excellent agreement with the result from the Dixon-Plot method (Fig. 3*E*, *insert*).

##### The Peptide hGW2052 Is a Specific Inhibitor for ATGL

**FIGURE 4.**
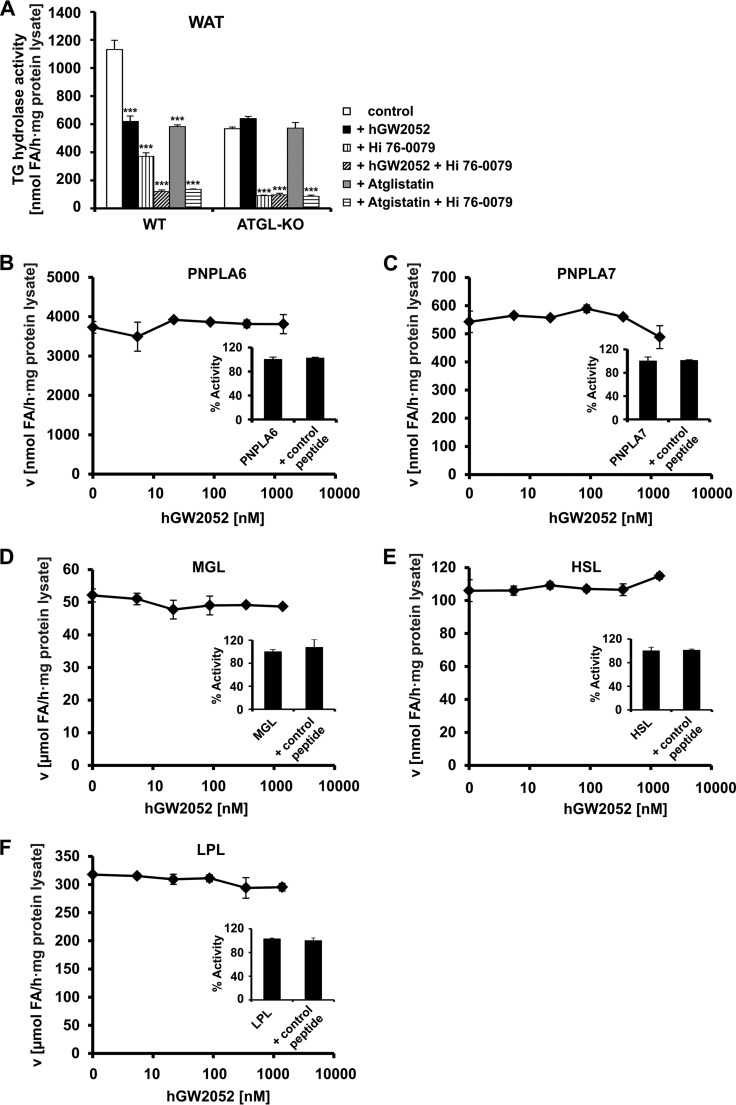
**The Peptide hGW2052 selectively inhibits ATGL.** TG hydrolase activity of ATGL, HSL, and lipoprotein lipase (*LPL*) was measured using triolein as substrate. MGL activity was determined using *rac*-(1,3)-monooleylglycerol as substrate. PNPLA6 and PNPLA7 activities were detected in the presence of 1-oleoyl-2-hydroxy-*sn*-glycero-3-phosphocholine. *A,* inhibition of TG hydrolase activity in WAT lysates of overnight fasted WT and ATGL-KO mice by 1.4 μm hGW2052, 40 μm Atglistatin, and 25 μm of the HSL inhibitor Hi 76-0079, respectively. Dose-dependent effect of hGW2052 and control peptide is shown on murine PNPLA6 (*B*), PNPLA7 lysophospholipase activities (*C*), murine MGL monoacylglycerol hydrolase activity (*D*), HSL triglyceride hydrolase activity (*E*), and bovine lipoprotein lipase triglyceride hydrolase activity (*F*).

Next, we investigated the specificity of the peptide hGW2052. ATGL and HSL together account for more than 95% of intracellular TG hydrolase activity in white adipose tissue (WAT) (48). For both enzymes, small molecule inhibitors are available. Atglistatin and Hi 76–0079 (N-methyl-phenyl carbamoyl triazole, Novo Nordisk, Denmark) act as specific inhibitors for ATGL and HSL, respectively (10, 48, 49). WAT lysates of WT and *Atgl*-KO mice were tested for TG hydrolase activity in the presence of Atglistatin, Hi 76–0079, and hGW2052. Addition of the ATGL inhibitors hGW2052 or Atglistatin reduced TG hydrolase activity of WAT lysates from WT animals by 45 and 49%, respectively. Addition of the HSL inhibitor Hi 76-0079 reduced TG hydrolase activity of WAT lysates by 67%. Combined addition of hGW2052 or Atglistatin (representing ATGL inhibitors) and Hi 76-0079 (representing an HSL inhibitor) reduced TG hydrolase activity of WAT by 89 and 88%, respectively (Fig. 4*A*). Neither hGW2052 nor Atglistatin inhibited WAT TG hydrolase activity derived from *Atgl-*KO mice (Fig. 4*A*). These results indicate that hGW2052 and Atglistatin specifically inhibit ATGL but not HSL activity.

As ATGL is a member of the patatin-like phospholipase domain containing protein (PNPLA) family (50), we further tested whether the synthetic peptide inhibits other proteins of the PNPLA family. As shown in Fig. 4, *B* and *C*, hGW2052 did not exert any inhibitory effect on the lysophospholipase activities of PLNPLA6 or PNPLA7, up to a concentration of 1.4 μm. As expected, the control peptide also did not affect the lysophospholipase activity of PNPLA6 and PNPLA7 (Fig. 4, *B* and *C*). Thus, we conclude that hGW2052 does not act as general inhibitor for members of the PNPLA protein family. We also tested for a potential effect of the inhibitory peptide hGW2052 on other extracellular (lipoprotein lipase) and intracellular (MGL and HSL) lipases. No inhibitory effect on any of these lipases was observed upon addition of up to 1.4 μm hGW2052 or control peptide, respectively (Fig. 4, *D–F*). Together, these experiments demonstrate that the peptide hGW2052 is not a general lipase inhibitor and, among the different lipases tested, rather acts specifically on ATGL.

## DISCUSSION

Abnormally high concentrations of circulating FAs have deleterious effects on the organism, including ectopic lipid accumulation, insulin resistance, inflammation, cellular dysfunction, and cell death (1, 2). ATGL catalyzes the hydrolysis of TG into diacylglycerols and FA, the first step in TG catabolism, and thus strongly affects FA concentrations in the circulation. Decreased ATGL activity exerts beneficial effects such as improved glucose tolerance and insulin sensitivity and protection from cancer-induced cachexia (8, 11, 51, 52). Therefore, it is of fundamental interest to understand the mechanisms that regulate ATGL-mediated lipolysis and concomitantly the release of potentially harmful FAs.

G0S2 has been identified to act as a physiological inhibitor of ATGL. However, very little is known about the molecular basis and the mode of ATGL inhibition by G0S2. Therefore, these questions were addressed in this study. We also identified minimal sequences of G0S2 that inhibit ATGL's TG hydrolase activity. Variants of G0S2 that were fully able to inhibit ATGL included extensive N- or C-terminal truncations of the wild-type protein (Tyr-27–Ser-103 and Met-1–Met-43, respectively). Interestingly, however, a combination of the identified N- and C-terminal boundaries (*e.g.* variants Lys-20–Met-43 or Tyr-27–Ala-52) did not result in G0S2 variants that were able to inhibit ATGL efficiently. We hypothesize that a core peptide Tyr-27–Met-43 is mainly involved in ATGL inhibition, yet a few additional residues are required to mediate full function of the inhibitory peptide.

Based on our results, we furthermore developed a short 34-amino acid peptide that acts as an efficient inhibitor of mouse and human ATGL. Inhibition kinetics performed in this study showed that the inhibitory peptide hGW2052 acts in a noncompetitive manner. Long-chain acyl-coenzyme A and Atglistatin are reported to inhibit ATGL upon interaction with the lipase (10, 13). Although noncompetitive inhibition is also reported for acyl-CoA, Atglistatin inhibits ATGL in a competitive manner (10, 13). Dose-response experiments revealed that wild-type G0S2 and the inhibitory peptide hGW2052 act in the nanomolar range (IC_50_ = 10 and 18 nm, respectively). Consequently, physiologically relevant ATGL regulation by G0S2 is very plausible, and administration of hGW2052 at physiological concentrations can be envisaged. This high affinity of G0S2 is in contrast to the affinity of oleoyl-CoA for ATGL, which exhibits a much higher IC_50_ value (33 μm) (13). Accordingly, the potential physiological relevance of an acyl-CoA-mediated feedback mechanism still remains to be elucidated. The small molecule Atglistatin exhibits an IC_50_ value in the low micromolar range, which further illustrates the potency of the inhibitory peptide.

To evaluate the specificity of the inhibitory peptide for ATGL, we analyzed the effect of hGW2052 on the activities of phospholipases, intracellular lipases, and extracellular lipases. ATGL belongs to the patatin domain containing family of proteins (PNPLA). Some family members have already been characterized and exhibit TG hydrolase activity (ATGL, also termed PNPLA2) or lysophospholipase activities (PNPLA6 and PNPLA7) (50). Although PNPLA family members show high sequence conservation within their common patatin domain, no effects on lysophospholipase activities of PNPLA6 or PNPLA7 could be observed upon incubation with the peptide hGW2052. Furthermore, our enzyme assays revealed that hGW2052 also does not inhibit HSL, MGL, or lipoprotein lipase activities and thus demonstrate high specificity for ATGL. Similar discrimination between general lipases, PNPLAs and ATGL was observed for Atglistatin, which was specifically developed and optimized to inhibit mouse ATGL (10). Long-chain acyl-CoAs, however, exhibit less specificity for lipase inhibition and have been reported to inhibit both ATGL and HSL (13, 53). It should be noted that the physiological roles of long-chain acyl-CoAs are diverse and include acyl-CoA-dependent activities of some PNPLA protein family members (54). Acyl-CoAs are highly abundant metabolic intermediates involved in multiple processes and thus clearly do not represent promising lead compounds for the development of ATGL inhibitors.

Genetic disruption of *Atgl* results in severe heart defects due to massive TG accumulation in cardiac muscle (8, 55). Thus, the application of a synthetic, competitive ATGL inhibitor, such as Atglistatin, as a therapeutic tool appears limited. Yet the single administration of Atglistatin to mice led to TG accumulation in liver but not the heart, accompanied by accumulation of the inhibitor in liver and adipose tissues (10). This demonstrates that a transient inhibition of ATGL is an effective means to inhibit TG catabolism and does not necessarily interfere with cardiac TG homeostasis. The peptide developed in this study harbors a high potential for selective inhibition of ATGL. Peptide inhibitors offer the great advantage that they can be fused with specific targeting sequences and as such allowing tissue-specific targeting (56–58), which could prevent harmful TG accumulation in the heart or other tissues. Competitive *versus* noncompetitive inhibition modes are additional important differences when comparing the potential impact of the peptide inhibitor hGW2052 with Atglistatin. hGW2052 inhibits ATGL in a noncompetitive manner and therefore inhibits ATGL despite high physiological substrate concentrations. Thus, hGW2052 represents a powerful research tool for tissue-selective inhibition of ATGL and has a high potential for translation into therapeutic application.

Structural knowledge on ATGL is very limited. It is predicted to exert lipolytic activity via a catalytic dyad that resides in the patatin domain within the N-terminal half of the lipase (7, 50). Previous reports demonstrated that G0S2 interacts with the N-terminal catalytic domain of ATGL (15). Here, we demonstrate that a G0S2-derived peptide does not compete with the TG substrate for ATGL binding. Similarly, inhibiting the truncated ATGL-289X variant by acyl-CoA showed that acyl-CoA binds to the N-terminal half of ATGL and is independent of substrate binding (13). In contrast, Atglistatin inhibits in a competitive mode indicating that the molecule probably binds within the substrate-binding pocket of ATGL. In this study, we further demonstrate that the inhibition of ATGL by the peptide hGW2052 works in the absence or presence of CGI-58. Similarly, ATGL inhibition by acyl-CoA and Atglistatin was reported to be independent of the presence of CGI-58 (10, 13). In brief, the currently available data of the ATGL interaction partners G0S2 and the inhibitory peptide, CGI-58, acyl-CoA, and Atglistatin point toward independent interaction regions, yet all are reported to reside within the N-terminal half of ATGL (15, 18, 20, 59). Unfortunately, no experimental three-dimensional structures for any of these players are known, which would aid in the identification of interaction surfaces. Furthermore, no mutations of ATGL are known that retain catalytic activity, but they lose the ability to interact with CGI-58 or G0S2. Clearly, more biochemical and structural knowledge is required to address these open questions.

## Footnotes

2 The abbreviations used are:

TG: triacylglycerol
ATGL: adipose triglyceride lipase
ATGL*: CGI-58 activated ATGL
HSL: hormone-sensitive lipase
MGL: monoacylglycerol lipase
PNPLA: patatin-like phospholipase domain containing protein
WAT: white adipose tissue
TCEP: tris(2-carboxyelthyl)phosphine hydrochloride
TEV: tobacco etch virus
FA: fatty acid
m: murine
h: human.

## References

[1] Li L. O. Klett E. L. Coleman R. A. (2010) Acyl-CoA synthesis, lipid metabolism and lipotoxicity. Biochim. Biophys. Acta 1801, 246–251 19818872 10.1016/j.bbalip.2009.09.024 PMC2824076

[2] Samuel V. T. Petersen K. F. Shulman G. I. (2010) Lipid-induced insulin resistance: unravelling the mechanism. Lancet 375, 2267–2277 20609972 10.1016/S0140-6736(10)60408-4 PMC2995547

[3] Unger R. H. Clark G. O. Scherer P. E. Orci L. (2010) Lipid homeostasis, lipotoxicity and the metabolic syndrome. Biochim. Biophys. Acta 1801, 209–214 19948243 10.1016/j.bbalip.2009.10.006

[4] Unger R. H. Scherer P. E. (2010) Gluttony, sloth and the metabolic syndrome: a roadmap to lipotoxicity. Trends Endocrinol. Metab. 21, 345–352 20223680 10.1016/j.tem.2010.01.009 PMC2880185

[5] Karpe F. Dickmann J. R. Frayn K. N. (2011) Fatty acids, obesity, and insulin resistance: time for a reevaluation. Diabetes 60, 2441–2449 21948998 10.2337/db11-0425 PMC3178283

[6] Mittendorfer B. Magkos F. Fabbrini E. Mohammed B. S. Klein S. (2009) Relationship between body fat mass and free fatty acid kinetics in men and women. Obesity 17, 1872–1877 19629053 10.1038/oby.2009.224 PMC3319738

[7] Zimmermann R. Strauss J. G. Haemmerle G. Schoiswohl G. Birner-Gruenberger R. Riederer M. Lass A. Neuberger G. Eisenhaber F. Hermetter A. Zechner R. (2004) Fat mobilization in adipose tissue is promoted by adipose triglyceride lipase. Science 306, 1383–1386 15550674 10.1126/science.1100747

[8] Haemmerle G. Lass A. Zimmermann R. Gorkiewicz G. Meyer C. Rozman J. Heldmaier G. Maier R. Theussl C. Eder S. Kratky D. Wagner E. F. Klingenspor M. Hoefler G. Zechner R. (2006) Defective lipolysis and altered energy metabolism in mice lacking adipose triglyceride lipase. Science 312, 734–737 16675698 10.1126/science.1123965

[9] Schweiger M. Lass A. Zimmermann R. Eichmann T. O. Zechner R. (2009) Neutral lipid storage disease: genetic disorders caused by mutations in adipose triglyceride lipase/PNPLA2 or CGI-58/ABHD5. Am. J. Physiol. Endocrinol. Metab. 297, E289–E296 19401457 10.1152/ajpendo.00099.2009

[10] Mayer N. Schweiger M. Romauch M. Grabner G. F. Eichmann T. O. Fuchs E. Ivkovic J. Heier C. Mrak I. Lass A. Höfler G. Fledelius C. Zechner R. Zimmermann R. Breinbauer R. (2013) Development of small-molecule inhibitors targeting adipose triglyceride lipase. Nat. Chem. Biol. 9, 785–787 24096302 10.1038/nchembio.1359 PMC3829776

[11] Das S. K. Eder S. Schauer S. Diwoky C. Temmel H. Guertl B. Gorkiewicz G. Tamilarasan K. P. Kumari P. Trauner M. Zimmermann R. Vesely P. Haemmerle G. Zechner R. Hoefler G. (2011) Adipose triglyceride lipase contributes to cancer-associated cachexia. Science 333, 233–238 21680814 10.1126/science.1198973

[12] Lass A. Zimmermann R. Oberer M. Zechner R. (2011) Lipolysis–a highly regulated multi-enzyme complex mediates the catabolism of cellular fat stores. Prog. Lipid Res. 50, 14–27 21087632 10.1016/j.plipres.2010.10.004 PMC3031774

[13] Nagy H. M. Paar M. Heier C. Moustafa T. Hofer P. Haemmerle G. Lass A. Zechner R. Oberer M. Zimmermann R. (2014) Adipose triglyceride lipase activity is inhibited by long-chain acyl-coenzyme A. Biochim. Biophys. Acta 1841, 588–594 24440819 10.1016/j.bbalip.2014.01.005 PMC3988850

[14] Lass A. Zimmermann R. Haemmerle G. Riederer M. Schoiswohl G. Schweiger M. Kienesberger P. Strauss J. G. Gorkiewicz G. Zechner R. (2006) Adipose triglyceride lipase-mediated lipolysis of cellular fat stores is activated by CGI-58 and defective in Chanarin-Dorfman Syndrome. Cell Metab. 3, 309–319 16679289 10.1016/j.cmet.2006.03.005

[15] Cornaciu I. Boeszoermenyi A. Lindermuth H. Nagy H. M. Cerk I. K. Ebner C. Salzburger B. Gruber A. Schweiger M. Zechner R. Lass A. Zimmermann R. Oberer M. (2011) The minimal domain of adipose triglyceride lipase (ATGL) ranges until leucine 254 and can be activated and inhibited by CGI-58 and G0S2, respectively. PLoS One 6, e26349 22039468 10.1371/journal.pone.0026349 PMC3198459

[16] Heckmann B. L. Zhang X. Xie X. Saarinen A. Lu X. Yang X. Liu J. (2014) Defective adipose lipolysis and altered global energy metabolism in mice with adipose overexpression of the lipolytic inhibitor G_0_/G_1_ switch gene 2 (G0S2). J. Biol. Chem. 289, 1905–1916 24302733 10.1074/jbc.M113.522011 PMC3900941

[17] Lu X. Yang X. Liu J. (2010) Differential control of ATGL-mediated lipid droplet degradation by CGI-58 and G0S2. Cell Cycle 9, 2719–2725 20676045 10.4161/cc.9.14.12181 PMC3040957

[18] Schweiger M. Paar M. Eder C. Brandis J. Moser E. Gorkiewicz G. Grond S. Radner F. P. Cerk I. Cornaciu I. Oberer M. Kersten S. Zechner R. Zimmermann R. Lass A. (2012) G0/G1 switch gene-2 regulates human adipocyte lipolysis by affecting activity and localization of adipose triglyceride lipase. J. Lipid Res. 53, 2307–2317 22891293 10.1194/jlr.M027409 PMC3466000

[19] Wang Y. Zhang Y. Qian H. Lu J. Zhang Z. Min X. Lang M. Yang H. Wang N. Zhang P. (2013) The G0/G1 switch gene 2 is an important regulator of hepatic triglyceride metabolism. PLoS One 8, e72315 23951308 10.1371/journal.pone.0072315 PMC3741160

[20] Yang X. Lu X. Lombès M. Rha G. B. Chi Y. I. Guerin T. M. Smart E. J. Liu J. (2010) The G_0_/G_1_ switch gene 2 regulates adipose lipolysis through association with adipose triglyceride lipase. Cell Metab. 11, 194–205 20197052 10.1016/j.cmet.2010.02.003 PMC3658843

[21] Yang X. Zhang X. Heckmann B. L. Lu X. Liu J. (2011) Relative contribution of adipose triglyceride lipase and hormone-sensitive lipase to tumor necrosis factor-α (TNF-α)-induced lipolysis in adipocytes. J. Biol. Chem. 286, 40477–40485 21969372 10.1074/jbc.M111.257923 PMC3220500

[22] Bächner D. Ahrens M. Schröder D. Hoffmann A. Lauber J. Betat N. Steinert P. Flohé L. Gross G. (1998) Bmp-2 downstream targets in mesenchymal development identified by subtractive cloning from recombinant mesenchymal progenitors (C3H10T1/2). Dev. Dyn. 213, 398–411 9853961 10.1002/(SICI)1097-0177(199812)213:4<398::AID-AJA5>3.0.CO;2-T

[23] Zandbergen F. Mandard S. Escher P. Tan N. S. Patsouris D. Jatkoe T. Rojas-Caro S. Madore S. Wahli W. Tafuri S. Müller M. Kersten S. (2005) The G0/G1 switch gene 2 is a novel PPAR target gene. Biochem. J. 392, 313–324 16086669 10.1042/BJ20050636 PMC1316267

[24] Choi H. Lee H. Kim T. H. Kim H. J. Lee Y. J. Lee S. J. Yu J. H. Kim D. Kim K. S. Park S. W. Kim J. W. (2014) G0/G1 switch gene 2 has a critical role in adipocyte differentiation. Cell Death Differ. 21, 1071–1080 24583640 10.1038/cdd.2014.26 PMC4207475

[25] Ma T. Lopez-Aguiar A. G. Li A. Lu Y. Sekula D. Nattie E. E. Freemantle S. Dmitrovsky E. (2014) Mice lacking G0S2 are lean and cold-tolerant. Cancer Biol. Ther. 15, 643–650 24556704 10.4161/cbt.28251 PMC4026087

[26] Zhang X. Xie X. Heckmann B. L. Saarinen A. M. Czyzyk T. A. Liu J. (2014) Targeted disruption of G0/G1 switch gene 2 enhances adipose lipolysis, alters hepatic energy balance, and alleviates high-fat diet-induced liver steatosis. Diabetes 63, 934–946 24194501 10.2337/db13-1422 PMC3931401

[27] Nielsen T. S. Møller N. (2014) Adipose triglyceride lipase and G0/G1 switch gene 2: approaching proof of concept. Diabetes 63, 847–849 24556865 10.2337/db13-1838

[28] Ma L. Robinson L. N. Towle H. C. (2006) ChREBP*Mlx is the principal mediator of glucose-induced gene expression in the liver. J. Biol. Chem. 281, 28721–28730 16885160 10.1074/jbc.M601576200

[29] Parikh H. Carlsson E. Chutkow W. A. Johansson L. E. Storgaard H. Poulsen P. Saxena R. Ladd C. Schulze P. C. Mazzini M. J. Jensen C. B. Krook A. Björnholm M. Tornqvist H. Zierath J. R. Ridderstråle M. Altshuler D. Lee R. T. Vaag A. Groop L. C. Mootha V. K. (2007) TXNIP regulates peripheral glucose metabolism in humans. PLoS Med. 4, e158 17472435 10.1371/journal.pmed.0040158 PMC1858708

[30] Nielsen T. S. Kampmann U. Nielsen R. R. Jessen N. Orskov L. Pedersen S. B. Jørgensen J. O. Lund S. Møller N. (2012) Reduced mRNA and protein expression of perilipin A and G0/G1 switch gene 2 (G0S2) in human adipose tissue in poorly controlled type 2 diabetes. J. Clin. Endocrinol. Metab. 97, E1348–E1352 22535977 10.1210/jc.2012-1159

[31] Yamada T. Park C. S. Burns A. Nakada D. Lacorazza H. D. (2012) The cytosolic protein G0S2 maintains quiescence in hematopoietic stem cells. PLoS One 7, e38280 22693613 10.1371/journal.pone.0038280 PMC3365016

[32] Yamada T. Park C. S. Shen Y. Rabin K. R. Lacorazza H. D. (2014) G0S2 inhibits the proliferation of K562 cells by interacting with nucleolin in the cytosol. Leuk. Res. 38, 210–217 24183236 10.1016/j.leukres.2013.10.006 PMC3946941

[33] Welch C. Santra M. K. El-Assaad W. Zhu X. Huber W. E. Keys R. A. Teodoro J. G. Green M. R. (2009) Identification of a protein, G0S2, that lacks Bcl-2 homology domains and interacts with and antagonizes Bcl-2. Cancer Res. 69, 6782–6789 19706769 10.1158/0008-5472.CAN-09-0128 PMC2841785

[34] Cristillo A. D. Heximer S. P. Russell L. Forsdyke D. R. (1997) Cyclosporin A inhibits early mRNA expression of G0/G1 switch gene 2 (G0S2) in cultured human blood mononuclear cells. DNA Cell Biol. 16, 1449–1458 9428793 10.1089/dna.1997.16.1449

[35] Koczan D. Guthke R. Thiesen H. J. Ibrahim S. M. Kundt G. Krentz H. Gross G. Kunz M. (2005) Gene expression profiling of peripheral blood mononuclear leukocytes from psoriasis patients identifies new immune regulatory molecules. Eur. J. Dermatol. 15, 251–257 16048752

[36] Nakamura N. Shimaoka Y. Tougan T. Onda H. Okuzaki D. Zhao H. Fujimori A. Yabuta N. Nagamori I. Tanigawa A. Sato J. Oda T. Hayashida K. Suzuki R. Yukioka M. Nojima H. Ochi T. (2006) Isolation and expression profiling of genes upregulated in bone marrow-derived mononuclear cells of rheumatoid arthritis patients. DNA Res. 13, 169–183 17082220 10.1093/dnares/dsl006

[37] Russell L. Forsdyke D. R. (1991) A human putative lymphocyte G0/G1 switch gene containing a CpG-rich island encodes a small basic protein with the potential to be phosphorylated. DNA Cell Biol. 10, 581–591 1930693 10.1089/dna.1991.10.581

[38] Kioka H. Kato H. Fujikawa M. Tsukamoto O. Suzuki T. Imamura H. Nakano A. Higo S. Yamazaki S. Matsuzaki T. Takafuji K. Asanuma H. Asakura M. Minamino T. Shintani Y. Yoshida M. Noji H. Kitakaze M. Komuro I. Asano Y. Takashima S. (2014) Evaluation of intramitochondrial ATP levels identifies G0/G1 switch gene 2 as a positive regulator of oxidative phosphorylation. Proc. Natl. Acad. Sci. U.S.A. 111, 273–278 24344269 10.1073/pnas.1318547111 PMC3890790

[39] Kusakabe M. Kutomi T. Watanabe K. Emoto N. Aki N. Kage H. Hamano E. Kitagawa H. Nagase T. Sano A. Yoshida Y. Fukami T. Murakawa T. Nakajima J. Takamoto S. Ota S. Fukayama M. Yatomi Y. Ohishi N. Takai D. (2010) Identification of G0S2 as a gene frequently methylated in squamous lung cancer by combination of in silico and experimental approaches. Int. J. Cancer 126, 1895–1902 19816938 10.1002/ijc.24947

[40] Kusakabe M. Watanabe K. Emoto N. Aki N. Kage H. Nagase T. Nakajima J. Yatomi Y. Ohishi N. Takai D. (2009) Impact of DNA demethylation of the G0S2 gene on the transcription of G0S2 in squamous lung cancer cell lines with or without nuclear receptor agonists. Biochem. Biophys. Res. Commun. 390, 1283–1287 19878646 10.1016/j.bbrc.2009.10.137

[41] Tokumaru Y. Yamashita K. Osada M. Nomoto S. Sun D. I. Xiao Y. Hoque M. O. Westra W. H. Califano J. A. Sidransky D. (2004) Inverse correlation between cyclin A1 hypermethylation and p53 mutation in head and neck cancer identified by reversal of epigenetic silencing. Cancer Res. 64, 5982–5987 15342377 10.1158/0008-5472.CAN-04-0993

[42] Gruber A. Cornaciu I. Lass A. Schweiger M. Poeschl M. Eder C. Kumari M. Schoiswohl G. Wolinski H. Kohlwein S. D. Zechner R. Zimmermann R. Oberer M. (2010) The N-terminal region of comparative gene identification-58 (CGI-58) is important for lipid droplet binding and activation of adipose triglyceride lipase. J. Biol. Chem. 285, 12289–12298 20164531 10.1074/jbc.M109.064469 PMC2852968

[43] Heier C. Taschler U. Rengachari S. Oberer M. Wolinski H. Natter K. Kohlwein S. D. Leber R. Zimmermann R. (2010) Identification of Yju3p as functional orthologue of mammalian monoglyceride lipase in the yeast *Saccharomyces cerevisiae*. Biochim. Biophys. Acta 1801, 1063–1071 20554061 10.1016/j.bbalip.2010.06.001 PMC2911655

[44] Kienesberger P. C. Lass A. Preiss-Landl K. Wolinski H. Kohlwein S. D. Zimmermann R. Zechner R. (2008) Identification of an insulin-regulated lysophospholipase with homology to neuropathy target esterase. J. Biol. Chem. 283, 5908–5917 18086666 10.1074/jbc.M709598200

[45] Schweiger M. Eichmann T. O. Taschler U. Zimmermann R. Zechner R. Lass A. (2014) Measurement of lipolysis. Methods Enzymol. 538, 171–193 24529439 10.1016/B978-0-12-800280-3.00010-4 PMC4018506

[46] Taschler U. Radner F. P. Heier C. Schreiber R. Schweiger M. Schoiswohl G. Preiss-Landl K. Jaeger D. Reiter B. Koefeler H. C. Wojciechowski J. Theussl C. Penninger J. M. Lass A. Haemmerle G. Zechner R. Zimmermann R. (2011) Monoglyceride lipase deficiency in mice impairs lipolysis and attenuates diet-induced insulin resistance. J. Biol. Chem. 286, 17467–17477 21454566 10.1074/jbc.M110.215434 PMC3093820

[47] Dixon M. (1953) The determination of enzyme inhibitor constants. Biochem. J. 55, 170–171 13093635 10.1042/bj0550170 PMC1269152

[48] Schweiger M. Schreiber R. Haemmerle G. Lass A. Fledelius C. Jacobsen P. Tornqvist H. Zechner R. Zimmermann R. (2006) Adipose triglyceride lipase and hormone-sensitive lipase are the major enzymes in adipose tissue triacylglycerol catabolism. J. Biol. Chem. 281, 40236–40241 17074755 10.1074/jbc.M608048200

[49] Ebdrup S. Sørensen L. G. Olsen O. H. Jacobsen P. (2004) Synthesis and structure-activity relationship for a novel class of potent and selective carbamoyl-triazole based inhibitors of hormone sensitive lipase. J. Med. Chem. 47, 400–410 14711311 10.1021/jm031004s

[50] Kienesberger P. C. Oberer M. Lass A. Zechner R. (2009) Mammalian patatin domain containing proteins: a family with diverse lipolytic activities involved in multiple biological functions. J. Lipid Res. 50, S63–S68 19029121 10.1194/jlr.R800082-JLR200 PMC2674697

[51] Hoy A. J. Bruce C. R. Turpin S. M. Morris A. J. Febbraio M. A. Watt M. J. (2011) Adipose triglyceride lipase-null mice are resistant to high-fat diet-induced insulin resistance despite reduced energy expenditure and ectopic lipid accumulation. Endocrinology 152, 48–58 21106876 10.1210/en.2010-0661

[52] Kienesberger P. C. Lee D. Pulinilkunnil T. Brenner D. S. Cai L. Magnes C. Koefeler H. C. Streith I. E. Rechberger G. N. Haemmerle G. Flier J. S. Zechner R. Kim Y. B. Kershaw E. E. (2009) Adipose triglyceride lipase deficiency causes tissue-specific changes in insulin signaling. J. Biol. Chem. 284, 30218–30229 19723629 10.1074/jbc.M109.047787 PMC2781577

[53] Severson D. L. Hurley B. (1984) Inhibition of the hormone-sensitive lipase in adipose tissue by long-chain fatty acyl coenzyme A. Lipids 19, 134–138 6323907 10.1007/BF02534504

[54] Kumari M. Schoiswohl G. Chitraju C. Paar M. Cornaciu I. Rangrez A. Y. Wongsiriroj N. Nagy H. M. Ivanova P. T. Scott S. A. Knittelfelder O. Rechberger G. N. Birner-Gruenberger R. Eder S. Brown H. A. Haemmerle G. Oberer M. Lass A. Kershaw E. E. Zimmermann R. Zechner R. (2012) Adiponutrin functions as a nutritionally regulated lysophosphatidic acid acyltransferase. Cell Metab. 15, 691–702 22560221 10.1016/j.cmet.2012.04.008 PMC3361708

[55] Hirano K. (2009) A novel clinical entity: triglyceride deposit cardiomyovasculopathy. J. Atheroscler. Thromb. 16, 702–705 19729869 10.5551/jat.1669

[56] Gray B. P. Brown K. C. (2014) Combinatorial peptide libraries: mining for cell-binding peptides. Chem. Rev. 114, 1020–1081 24299061 10.1021/cr400166n PMC4053476

[57] Hossen M. N. Kajimoto K. Akita H. Hyodo M. Ishitsuka T. Harashima H. (2010) Ligand-based targeted delivery of a peptide modified nanocarrier to endothelial cells in adipose tissue. J. Control Release 147, 261–268 20647023 10.1016/j.jconrel.2010.07.100

[58] Kolonin M. G. Saha P. K. Chan L. Pasqualini R. Arap W. (2004) Reversal of obesity by targeted ablation of adipose tissue. Nat. Med. 10, 625–632 15133506 10.1038/nm1048

[59] Schweiger M. Schoiswohl G. Lass A. Radner F. P. Haemmerle G. Malli R. Graier W. Cornaciu I. Oberer M. Salvayre R. Fischer J. Zechner R. Zimmermann R. (2008) The C-terminal region of human adipose triglyceride lipase affects enzyme activity and lipid droplet binding. J. Biol. Chem. 283, 17211–17220 18445597 10.1074/jbc.M710566200

